# Prediction of preterm birth in nulliparous women using logistic regression and machine learning

**DOI:** 10.1371/journal.pone.0252025

**Published:** 2021-06-30

**Authors:** Reza Arabi Belaghi, Joseph Beyene, Sarah D. McDonald

**Affiliations:** 1 Department of Obstetrics and Gynecology, McMaster University, Hamilton, Ontario, Canada; 2 Department of Statistics, University of Tabriz, Tabriz, Iran; 3 Department of Health Research Methods, Evidence & Impact, McMaster University, Hamilton, Ontario, Canada; 4 Department of Mathematics and Statistics, McMaster University, Hamilton, Ontario, Canada; 5 Department of Obstetrics and Gynecology (Division of Maternal-Fetal Medicine), McMaster University, Hamilton, Ontario, Canada; 6 Department of Radiology, McMaster University, Hamilton, Ontario, Canada; Holbaek Sygehus, DENMARK

## Abstract

**Objective:**

To predict preterm birth in nulliparous women using logistic regression and machine learning.

**Design:**

Population-based retrospective cohort.

**Participants:**

Nulliparous women (N = 112,963) with a singleton gestation who gave birth between 20–42 weeks gestation in Ontario hospitals from April 1, 2012 to March 31, 2014.

**Methods:**

We used data during the first and second trimesters to build logistic regression and machine learning models in a “training” sample to predict overall and spontaneous preterm birth. We assessed model performance using various measures of accuracy including sensitivity, specificity, positive predictive value, negative predictive value, and area under the receiver operating characteristic curve (AUC) in an independent “validation” sample.

**Results:**

During the first trimester, logistic regression identified 13 variables associated with preterm birth, of which the strongest predictors were diabetes (Type I: adjusted odds ratio (AOR): 4.21; 95% confidence interval (CI): 3.23–5.42; Type II: AOR: 2.68; 95% CI: 2.05–3.46) and abnormal pregnancy-associated plasma protein A concentration (AOR: 2.04; 95% CI: 1.80–2.30). During the first trimester, the maximum AUC was 60% (95% CI: 58–62%) with artificial neural networks in the validation sample. During the second trimester, 17 variables were significantly associated with preterm birth, among which complications during pregnancy had the highest AOR (13.03; 95% CI: 12.21–13.90). During the second trimester, the AUC increased to 65% (95% CI: 63–66%) with artificial neural networks in the validation sample. Including complications during the pregnancy yielded an AUC of 80% (95% CI: 79–81%) with artificial neural networks. All models yielded 94–97% negative predictive values for spontaneous PTB during the first and second trimesters.

**Conclusion:**

Although artificial neural networks provided slightly higher AUC than logistic regression, prediction of preterm birth in the first trimester remained elusive. However, including data from the second trimester improved prediction to a moderate level by both logistic regression and machine learning approaches.

## Introduction

Preterm birth (PTB), birth before 37 weeks, is the leading cause of neonatal death and disability [1]. Approximately, 50% of all perinatal deaths are caused by PTB [2]. In the U.S., almost 10% of babies are born preterm [3], costing the healthcare system at least $26 billion yearly [4]. In Canada, PTB comprises 8% of all births and results in direct costs of $580 million annually [5]. Risk factors for PTB are heterogeneous and include previous PTB, race, age, nulliparity, urinary tract infection, smoking, and bleeding during early pregnancy [6–8]. Prediction of PTB would facilitate the use of therapeutic interventions to reduce infant morbidity and mortality, thereby benefitting families, society, and the healthcare system.

Previous studies have found the prediction of PTB to be challenging, whether by logistic regression or machine learning. The area under the receiver operating characteristic curve (AUC) for prediction of PTB in previous studies ranged from 62% to 72% depending on the number of predictors and study design [9–15]. The predictive power of the machine learning model developed by Fergus *et al*. [16] was promising (AUC, 95%), but measuring uterine electrical signals (electrohysterography) is not practical on a large scale. Another drawback was the synthetic oversampling of the whole dataset, rather than just the training dataset, thereby calling into question the 95% AUC of that work.

Machine learning is a computer programming approach whereby computers learn from “big data” to make better predictions [17]. In 2019, machine learning was identified as one of the most advanced tools for prenatal diagnosis [18]. Morover, machine learning has been broadly applied in medicine, from cancer detection [19, 20] to prediction of cardiovascular diseases [21], among others. In this study, we considered some of state-of-the-art machine learning methods, including decision trees, random forests, and artificial neural networks, that are frequently used in medicine to develop prediction models [21–28]. We also considered logistic regression as a traditional statistical approach to develop prediction models [29]. Unlike logistic regression, machine learning approaches are free of statistical assumptions (such as linearity and uncorrelated predictors) and can handle complex interactions between predictive factors without these interactions being explicitly specified [27, 30].

We aimed to overcome the challenges of predicting PTB, especially for nulliparous women, by evaluating logistic regression and multiple machine learning algorithms. To this end, we considered variables available in clinical care, including some not previously assessed in other studies. Our study aimed to: 1) identify important predictors associated with PTB during the first and second trimester in nulliparous women from a large population cohort; and 2) construct models to predict PTB based on logistic regression and robust machine learning algorithms.

## Methods and materials

### Data and population

Ontario comprises 40% of the Canadian population and has approximately 140,000 births each year [31]. We performed a population-based retrospective cohort study using Ontario’s Better Outcomes Registry and Network (BORN) database, which includes a wide range of maternal, antenatal, and birth data [32]. We included all nulliparous women with singleton births who gave birth between 20 and 42 weeks gestation in an Ontario hospital between April 1, 2012 and March 31, 2014.

#### Outcome

PTB was the primary outcome variable in this study, defined as gestational age at birth (from ultrasound estimation or calculation from the first day of the last menstrual period) <37 weeks. We also considered spontaneous PTB as a secondary outcome. Spontaneous PTB was identified using the definition of Maghsouldu *et al*. [33], i.e.: not “induced”, not “caesarean section” and not “augmented labor”.

#### Predictors

We considered predictors based on our literature review of PTB risk factors during the first and second trimesters [7, 34]. We considered socio-demographic variables including maternal age, height, pre-pregnancy body mass index (BMI), gestational weight gain during the first trimester, income, education, race, and immigration status. Further, we included the number of previous abortions (which includes miscarriages), conception type, smoking status, alcohol consumption, folic acid use, pre-existing medical health conditions, diabetes, pre-existing mental health conditions (such as anxiety, depression, and addiction) and antenatal health care provider type.

Pregnancy-associated plasma protein A and free beta-subunit of human chorionic gonadotropin were measured during the first trimester as part of the screen for Down syndrome [30], but we considered them as potential markers of placental and preeclamptic diseases [35]. We also included ultrasound measurement of nuchal translucency as another predictor [36]. For the second-trimester models, we included all of the predictors from the first trimester plus information that became available during the second trimester including dimeric inhibin A, unconjugated estriol, human chorionic gonadotropin, alpha-fetoprotein concentration, hypertensive disorders of pregnancy, gestational diabetes, infections, medication exposure, sex of the fetus, and complications during pregnancy [37].

We grouped maternal height into four categories, including <150 cm, 150 cm—169 cm, 160 cm—169 cm, and ≥170 cm. We classified pre-pregnancy BMI as underweight (<18.5 kg/m^2^), normal weight (18.5–24.9 kg/m^2^), overweight (25–29.9 kg/m^2^), and obese (≥30 kg/m^2^), according to World Health Organization criteria [38, 39]. We used the Institute of Medicine guidelines [40] to categorize gestational weight gain into three groups, including recommended weight gain, less than recommended weight gain, and more than recommended weight gain. For income, education, race, and immigration status, we used neighbourhood income quartiles, neighbourhood education quartiles, neighbourhood immigrant concentration, and neighbourhood minority quartiles, respectively (see S1 Table for the definition of these variables).

We categorized the number of previous abortions (including spontaneous and therapeutic abortions) into four groups based on Oliver *et al*. [41], including 0, 1, 2, and 3+. We grouped the pre-existing health conditions variable in the BORN database into “Yes” or “No” since that variable had more than 1000 possible entries (S2 Table). We treated pre-existing mental health conditions (S3 Table) as a binary categorical variable. We classified the conception type into: spontaneous, *in vitro* fertilization (IVF, or a combination of IVF and other methods), and other methods (such as Surrogate, Intrauterine insemination alone, or unknown) [42].

We classified protein concentrations (pregnancy-associated plasma protein A, free beta-subunit of human chorionic gonadotropin, dimeric inhibin A, unconjugated estriol, human chorionic gonadotropin, and alpha-fetoprotein) and nuchal translucency as normal, abnormal, and missing (cut-off values shown in S4 Table). The variable “complications during pregnancy” had more than 600 categories, and we therefore categorized data for this variable into three groups based on maternal-fetal expertise (SDM) as follows: no complications, mild-moderate complications, and severe complications [37].

### Statistical analysis

We used the Chi-square test and univariate logistic regression to measure associations between predictors and PTB. We assessed statistical significance using 2-sided p-values, with a p-value <0.05 considered statistically significant. We then proceed with variable selection using stepwise multivariable logistic regression based on the Akaike Information Criterion (AIC). We also utilized the Boruta algorithm to select important variables for the machine learning models [43]. In short, Boruta is based on the random forest machine learning method, which selects relevant variables that significantly impact the prediction power of the model [43].

We followed the guidelines for the Transparent Reporting of a Multivariable Prediction Model for Individual Prognosis or Diagnosis [44] for establishing prediction models. Based on these guidelines, we selected 2/3 of the data as the training set and the remaining 1/3 of the data as the test (validation) set. We balanced the training samples using a random over-sampling technique [45]. We then used ten-fold cross-validation to establish machine learning models. Finally, we used the test data to evaluate the performance of the proposed prediction models by comparing the sensitivity, specificity, positive predictive values, negative predictive values, and AUC. We performed all machine learning computations in *R* software using the *caret* package [46].

We applied multiple imputation with 10 imputations [47–49] to replace missing observations on the predictors. However, for plasma proteins and nuchal translucency, missing data were treated as a new category since a large proportion of women chose not to enroll in screening for Down syndrome. We also treated gestational weight gain during the first trimester in a similar manner, since the lack of recording of weight gain may reflect less than optimal care. The Hamilton Integrated Research Ethics Board approved the study before study commencement (approval #: 14-714-C).

## Results

### Study participants and univariate analysis

Of 112,963 nulliparous women with singleton pregnancies, PTB occurred in 6,955 (6.2%, Table 1). Out of all PTBs, there were 3,695 (53%) spontaneous PTBs. Approximately 5% of patients were younger than 20 years of age, while 13% were over age 35 years. Approximately 2% of patients had three or more previous abortions including miscarriages. More than 50% of patients had a non-ideal pre-pregnancy BMI, of which 17.34% and 12.58% were overweight and obese, respectively. Approximately 17% of the cohort had at least one pre-existing medical condition. Only 78.67% of the patients had a documented first-trimester appointment.

**Table 1:** Distribution of maternal baseline characteristics, demographics, and clinical variables in nulliparous women.

Variables	Levels	N	%
Age (years)	<20	5782	5.12
	20–24	17979	15.92
	25–29	36309	32.14
	30–34	34798	30.80
	35+	14817	13.12
	Missing	3278	2.90
Height	<150 cm	2663	2.36
	150 cm-159 cm	21714	19.22
	160 cm-169 cm	51090	45.23
	≥170 cm	22662	20.06
	Missing	14834	13.13
	Mean = 163.7, SD = 7.34		
Pre-pregnancy body mass index (kg/m^2^)	Normal	51225	45.35
	Overweight	19584	17.34
	Obese	14212	12.58
	Underweight	5929	5.25
	Missing	22013	19.49
	Mean = 24.9, SD = 6.29		
Neighbourhood income quartile	First quartile (lowest)	29891	26.46
	Second quartile	25117	22.23
	Third quartile	26122	23.12
	Fourth quartile (highest)	27466	24.31
	Missing	4367	3.87
Neighbourhood education quartile	First quartile (lowest)	27849	24.65
	Second quartile	28552	25.28
	Third quartile	28089	24.87
	Fourth quartile (highest)	24980	22.11
	Missing	3493	3.09
Neighbourhood minority quartile	First quartile (lowest)	23762	21.04
	Second quartile	18718	16.57
	Third quartile	23705	20.98
	Fourth quartile (highest)	43285	38.32
	Missing	3493	3.09
Neighbourhood immigration quartile	First quartile (lowest)	24129	21.36
	Second quartile	20274	17.95
	Third quartile	24785	21.94
	Fourth quartile (highest)	39937	35.35
	Missing	3838	3.40
Smoking status	Non-smoker	97265	86.10
	Smoker	10986	9.73
	Missing	4712	4.17
Ex-smoker	No	71466	63.26
	Yes	16153	14.30
	Missing	25344	22.44
Alcohol consumption	No	101902	90.21
	Yes	2185	1.93
	Missing	8876	7.86
Drug (substance) use	No	102688	90.90
	Yes	2555	2.26
	Missing	7720	6.83
First-trimester visit	Yes	88866	78.67
	No	10983	9.72
	Unknown	13114	11.61
Antenatal health care provider	Obstetrician	98471	87.17
	Midwife	13561	12.00
	Missing	931	0.82
Folic acid use	Yes	78617	69.60
	No	21199	18.77
	Missing	13147	11.64
Intention to breastfeed	Yes	101057	89.46
	No	4933	4.37
	Missing	6973	6.17
Pre-existing health conditions	No	88390	78.25
	Yes	19608	17.36
	Missing	4965	4.40
Pre-existing mental health conditions	No	91666	81.15
	Yes	14932	13.22
	Missing	7720	6.83
Number of previous abortions (including miscarriages)	0	80615	71.36
	1	19189	16.99
	2	5334	4.72
	3+	2299	2.04
	Missing	5526	4.89
Conception type	Spontaneous	105061	93.00
	IVF and combination	2176	1.93
	Other	2662	2.36
	Missing	3064	2.71
Gravidity	Mean = 1.38, SD = 0.84		
Diabetes	No diabetes	102308	90.57
	Type I	356	0.32
	Type II	454	0.40
	Missing	9845	8.72
Gestational weight gain during the first trimester	Recommended	10034	8.88
	<Recommended	20477	18.13
	>Recommended	18842	16.68
	Missing	63610	56.31
Pregnancy-associated plasma protein A	Normal	60121	53.22
	Abnormal	3126	2.77
	Missing	49716	44.01
Free beta-subunit of human chorionic gonadotropin	Normal	105928	93.77
	Abnormal	6350	5.62
	Missing	685	0.61
Nuchal translucency	Normal	50550	44.75
	Abnormal	47	0.04
	Missing	62366	55.21
Dimeric inhibin A	Normal	7746	6.86
	Abnormal	564	0.50
	Missing	104653	92.64
Unconjugated estriol	Normal	61445	54.39
	Abnormal	290	0.26
	Missing	51228	45.35
Human chorionic gonadotropin	Normal	60733	53.76
	Abnormal	899	0.80
	Missing	51331	45.44
Alpha-fetoprotein	Normal	60610	53.65
	Abnormal	1616	1.44
	Missing	50737	44.9
Diabetes during the second trimester	No diabetes	97048	85.91
	Gestational diabetes	5228	4.63
	Type I	356	0.32
	Type II	454	0.40
	Type unknown	32	0.03
	Missing	9845	8.72
Hypertensive disorder	None	99619	88.19
	Eclampsia	63	0.06
	Gestational hypertension	5267	4.66
	HELLP	179	0.16
	Preeclampsia	914	0.81
	Unknown	6921	6.13
Infection(s)	No	80156	70.96
	Yes	24697	21.86
	Missing	8110	7.18
Medication exposure	No	20743	18.36
	Vitamin and herbals	50410	44.63
	Other medication	30384	26.90
	Missing	11426	10.11
Sex of fetus	Female	54612	48.35
	Male	58065	51.40
	Missing	286	0.25
Complications during pregnancy	No complications	90302	79.94
	Mild-moderate complications	4676	4.14
	Severe complications	14255	12.62
	Missing	3730	3.30

Preterm birth: n = 6,955 (6.16%); Spontaneous PTB: n = 3695 (5.62%); Term birth: n = 106,008 (93.84%); SD: Standard deviation; IVF: *In vitro* fertilization; Pre-existing maternal health conditions shown in S2 Table. Pre-existing mental health conditions shown in S3 Table.

During the first trimester, we examined 23 predictors (Table 2). Women who were under 25 years of age, shorter in stature (<160 cm), had pre-pregnancy obesity, conceived with IVF, had prior medical conditions including diabetes, and those with low pregnancy-associated plasma protein A concentrations were more likely than women without these conditions to experience PTB. During the second trimester, we examined 35 predictors of PTB. Women who were over 29 years of age, had abnormal concentrations of the assessed proteins, diabetes, hypertensive disorders of pregnancy, women carrying male fetuses, and those with pregnancy complications were more likely than women without these conditions to experience PTB (Table 3).

**Table 2:** Univariate analyses of associations between each predictor and preterm birth during the first trimester in nulliparous women.

		Term birth		Preterm birth		Chi-square test		
		85457 (93.8%)		5645 (6.2%)				
Variables	Levels	N	%	N	%	P-Value	Crude OR	95% CI
Age (years)	<20	4149	4.86	223	3.95	<0.001	1.20	(1.05–1.39)
	20–24	13874	16.24	791	14.01		1.24	(1.04–1.24)
	25–29	29361	34.36	1908	33.80		Reference	
	30–34	27310	31.96	1897	33.60		0.93	(0.87–0.99)
	35+	10763	12.59	826	14.63		0.84	(0.77–0.92)
Height	<150 cm	1963	2.30	172	3.05	<0.001	1.33	(1.13–1.55)
	150 cm-159 cm	17588	20.58	1395	24.71		1.20	(1.12–1.29)
	160 cm-169 cm	46763	54.72	3085	54.65		Reference	
	≥170 cm	19143	22.40	993	17.59		0.79	(0.73–0.84)
Pre-pregnancy body mass index (kg/m^2^)	Normal	52107	60.97	3245	57.48	<0.001	Reference	
	Overweight	16434	19.23	1103	19.54		1.07	(1.00–1.15)
	Obese	12315	14.41	983	17.41		1.28	(1.18–1.38)
	Underweight	4601	5.38	314	5.56		1.09	(0.97–1.23)
Neighbourhood income quartile	First quartile (lowest)	22363	26.17	1481	26.24	0.87	0.98	(0.91–1.06)
	Second quartile	19930	23.32	1341	23.76		Reference	
	Third quartile	21431	25.08	1401	24.82		0.97	(0.90–1.05)
	Fourth quartile (highest)	21733	25.43	1422	25.19		0.97	(0.90–1.04)
Neighbourhood education quartile	First quartile (lowest)	20734	24.26	1302	23.06	0.029	0.98	(0.90–1.05)
	Second quartile	23152	27.09	1490	26.40		Reference	
	Third quartile	22149	25.92	1493	26.45		1.04	(0.97–1.12)
	Fourth quartile (highest)	19422	22.73	1360	24.09		1.08	(1.01–1.17)
Neighbourhood minority quartile	First quartile (lowest)	20505	23.99	1415	25.07	0.048	1.01	(0.93–1.09)
	Second quartile	15694	18.36	1071	18.97		Reference	
	Third quartile	17916	20.96	1186	21.01		0.97	(0.89–1.05)
	Fourth quartile (highest)	31342	36.68	1973	34.95		0.92	(0.85–0.99)
Neighbourhood immigration quartile	First quartile (lowest)	21124	24.72	1518	26.89	0.001	1.11	(1.02–1.20)
	Second quartile	16978	19.87	1098	19.45		Reference	
	Third quartile	18742	21.93	1253	22.20		1.03	(0.95–1.12)
	Fourth quartile (highest)	28613	33.48	1776	31.46		0.95	(0.88–1.03)
Ex-smoker	No	70981	83.06	4632	82.05	0.054	Reference	
	Yes	14476	16.94	1013	17.95		1.07	(0.99–1.14)
Smoking status	Non-smoker	76892	89.98	5017	88.88	0.008	Reference	
	Smoker	8565	10.02	628	11.12		1.12	(1.03–1.22)
Folic acid use	Yes	68486	80.14	4610	81.67	0.006	Reference	
	No	16971	19.86	1035	18.33		0.90	(0.84–0.97)
Conception type	Spontaneous	81713	95.62	5276	93.46	<0.001	Reference	
	*In vitro* fertilization and combination	1536	1.80	204	3.61		2.07	(1.76–2.38)
	Other	2208	2.58	165	2.92		1.15	(0.98–1.35)
Number of previous abortions	0	64133	75.05	4113	72.86	<0.001	Reference	
	1	15254	17.85	1048	18.57		1.07	(0.99–1.14)
	2	4268	4.99	313	5.54		1.14	(1.01–1.28)
	3+	1802	2.11	171	3.03		1.48	(1.25–1.73)
Gravidity		Mean = 1.39, SD = 0.83		Mean = 1.45, SD = 0.93		<0.001	1.07	(1.05–1.11)
Gestational weight gain during the first trimester	Recommended	7934	9.28	533	9.44	0.053	Reference	
	>Recommended	14535	17.01	1036	18.35		1.07	(0.95–1.18)
	<Recommended	16107	18.85	1059	18.76		0.98	(0.87–1.09)
	Missing	46881	54.86	3017	53.45		0.96	(0.87–1.05)
Antenatal health care provider	Obstetrician	73694	86.24	5104	90.42	<0.001	Reference	
	Midwife	11763	13.76	541	9.58		0.66	(0.60–0.72)
Alcohol consumption	No	83881	98.16	5539	98.12	0.896	Reference	
	Yes	1576	1.84	106	1.88		1.02	(0.83–1.25)
Drug (substance) use	No	83660	97.90	5470	96.90	<0.001	Reference	
	Yes	1797	2.10	175	3.10		1.48	(1.26–1.74)
Pre-existing health conditions	None	70541	82.55	4259	75.45	<0.001	Reference	
	Yes	14916	17.45	1386	24.55		1.53	(1.44–1.63)
Pre-existing mental health conditions	No	73626	86.16	4720	83.61	<0.001	Reference	
	Yes	11831	13.84	925	16.39		1.21	(1.13–1.31)
Diabetes during the first trimester	No diabetes	84938	99.39	5480	97.08	<0.001	Reference	
	Type I	226	0.26	86	1.52		5.90	(4.27–7.53)
	Type II	293	0.34	79	1.40		4.17	(3.23–5.33)
Pregnancy-associated plasma protein A	Normal	46161	54.02	3049	54.01	<0.001	Reference	
	Abnormal	2215	2.59	324	5.74		2.21	(1.96–2.50)
	Missing	37081	43.39	2272	40.25		0.93	(0.87–0.98)
Nuchal translucency	Normal	47496	55.58	3323	58.87	<0.001	Reference	
	Abnormal	124	0.15	8	0.14		0.92	(0.41–1.76)
	Missing	37837	44.28	2314	40.99		0.87	(0.92–0.92)
Free beta-subunit of human chorionic gonadotropin	Normal	3665	4.29	254	4.50	0.249	Reference	
	Abnormal	396	0.46	34	0.60		1.23	(0.83–1.77)
	Missing	81396	95.25	5357	94.90		0.94	(0.85–1.08)

SD: Standard deviation; IVF: *In vitro* fertilization; Pre-existing maternal health conditions shown in S2 Table. Pre-existing mental health conditions shown in S3 Table.

**Table 3:** Univariate analyses of associations between each predictor and preterm birth during the second trimester in nulliparous women.

		Term birth		Preterm birth		Chi-square test		
		108905 (93.4%)		7754 (6.6%)				
Variables	Levels	N	%	N	%	P-values	OR	95% CI
Age (years)	<20	5696	5.23	322	4.15	<0.001	0.81	(0.72–0.91)
	20–24	17681	16.24	1115	14.38		0.90	(0.84–0.97)
	25–29	36048	33.10	2505	32.31		Reference	
	30–34	34813	31.97	2598	33.51		1.07	(1.01–1.13)
	35+	14667	13.47	1214	15.66		1.19	(1.10–1.28)
Height	<150 cm	2557	2.35	232	2.99	<0.001	1.28	(1.10–1.46)
	150 cm—159 cm	22590	20.74	1907	24.59		1.18	(1.12–1.26)
	160 cm—169 cm	60107	55.19	4270	55.07		Reference	
	≥170 cm	23651	21.72	1345	17.35		0.78	(0.73–0.84)
Pre- pregnancy BMI (kg/m^2^)	Normal	68198	62.62	4646	59.92	<0.001	Reference	
	Overweight	20226	18.57	1475	19.02		1.07	(1.00–1.14)
	Obese	14648	13.45	1218	15.71		1.22	(1.14–1.30)
	Underweight	5833	5.36	415	5.35		1.04	(0.94–1.15)
Neighbourhood income quartile	First quartile (lowest)	30047	27.59	2182	28.14	0.350	1.01	(0.92–1.06)
	Second quartile	25068	23.02	1806	23.29		Reference	
	Third quartile	26142	24.00	1866	24.06		0.99	(0.90–1.05)
	Fourth quartile (highest)	27648	25.39	1900	24.50		0.95	(0.89–1.01)
Neighbourhood education quartile	First quartile (lowest)	27948	25.66	1878	24.22	0.020	0.94	(0.88–1.01)
	Second quartile	28630	26.29	2027	26.14		Reference	
	Third quartile	27684	25.42	2012	25.95		1.02	(0.96–1.12)
	Fourth quartile (highest)	24643	22.63	1837	23.69		1.05	(0.98–1.12)
Neighbourhood minority quartile	First quartile (lowest)	23348	21.44	1709	22.04	0.500	1.01	(0.94–1.09)
	Second quartile	18283	16.79	1317	16.98		Reference	
	Third quartile	23105	21.22	1608	20.74		0.96	(0.91–1.04)
	Fourth quartile (highest)	44169	40.56	3120	40.24		0.98	(0.91–1.04)
Neighbourhood immigration quartile	First quartile (lowest)	24099	22.13	1822	23.50	0.040	1.09	(1.01–1.17)
	Second quartile	19780	18.16	1366	17.62		Reference	
	Third quartile	24219	22.24	1683	21.70		1.01	(0.93–1.09)
	Fourth quartile (highest)	40807	37.47	2883	37.18		1.02	(0.95–1.02)
Smoking status	Non-smoker	98461	90.41	6906	89.06	<0.001	Reference	
	Smoker	10444	9.59	848	10.94		1.15	(1.07–1.24)
Ex-smoker	No	91890	84.38	6479	83.56	0.060	Reference	
	Yes	17015	15.62	1275	16.44		1.06	(0.99–1.13)
Alcohol consumption	No	106830	98.09	7590	97.88	0.210	Reference	
	Yes	2075	1.91	164	2.12		1.02	(0.93–1.30)
Drug (substance) use	No	106518	97.81	7490	96.60	<0.001	Reference	
	Yes	2387	2.19	264	3.40		1.48	(1.37–1.78)
Number of previous abortions	0	82064	75.35	5601	72.23	<0.001	Reference	
	1	18748	17.22	1409	18.17		1.10	(1.03–1.16)
	2	5573	5.12	455	5.87		1.19	(1.08–1.31)
	3+	2520	2.31	289	3.73		1.68	(1.48–1.90)
Gravidity		Mean = 1.42,		Mean = 1.52,		<0.000	1.11	(1.0591.14)
		SD = 0.84		SD = 0.96				
Gestational weight gain during the first trimester	Recommended	9604	8.82	686	8.85	0.070	Reference	
	>Recommended	17942	16.47	1344	17.33		1.05	(0.95–1.15)
	<Recommended	19556	17.96	1317	16.98		0.94	(0.85–1.04)
	Missing	61803	56.75	4407	56.84		0.99	(0.91–1.08)
Antenatal health care provider	Obstetrician	95470	87.66	7122	91.85	<0.001		
	Midwife	13435	12.34	632	8.15		0.63	(0.58–0.68)
Diabetes	No diabetes	108260	99.41	7523	97.02	<0.001	Reference	
	Type I	269	0.25	123	1.59		6.58	(5.29–8.13)
	Type II	376	0.35	108	1.39		4.13	(3.31–5.10)
Pre-existing health conditions	No	94116	86.42	6473	83.48	<0.001	Reference	
	Yes	14789	13.58	1281	16.52		1.26	(1.18–1.34)
Pre-existing mental health conditions	None	90395	83.00	5879	75.82	<0.001	Reference	
	Yes	18510	17.00	1875	24.18		1.56	(1.47–1.64)
Folic acid use	Yes	85553	78.56	6118	78.90	0.490	Reference	
	No	23352	21.44	1636	21.10		0.98	(0.92–1.03)
Conception type	Spontaneous	104362	95.83	7293	94.05	<0.001	Reference	
	IVF or combination	2008	1.84	264	3.40		1.88	(1.64–2.13)
	Other	2535	2.33	197	2.54		1.11	(0.95–1.28)
Pregnancy-associated plasma protein-A	Normal	58076	53.33	4122	53.16	<0.001	Reference	
	Abnormal	2792	2.56	472	6.09		2.38	(2.14–2.63)
	Missing	48037	44.11	3160	40.75		0.92	(0.88–0.97)
Nuchal translucency	Normal	59980	55.08	4539	58.54	<0.001	Reference	
	Abnormal	158	0.15	18	0.23		1.50	(0.89–2.38)
	Missing	48767	44.78	3197	41.23		0.86	(0.82–0.90)
Free beta-subunit of human chorionic gonadotropin	Normal	6195	5.69	468	6.04	0.300	Reference	
	Abnormal	670	0.62	54	0.70		1.07	(0.78–1.41)
	Missing	102040	93.70	7232	93.27		0.93	(0.88–1.03)
First trimester visit	Yes	85457	78.47	5645	72.80	<0.001	Reference	
	No	10433	9.58	742	9.57		1.07	(0.99–1.16)
	Unknown	13015	11.95	1367	17.63		1.59	(1.50–1.69)
Intention to breastfeed	Yes	4514	4.14	549	7.08	<0.001		
	No	104391	95.86	7205	92.92		1.76	(1.60–1.92)
Dimeric inhibin A	Normal	7415	6.81	535	6.90	<0.001	Reference	
	Abnormal	516	0.47	63	0.81		1.69	(1.27–2.21)
	Missing	100974	92.72	7156	92.29		0.98	(0.89–1.07)
Unconjugated estriol	Normal	59024	54.20	4440	57.26	<0.001	Reference	
	Abnormal	256	0.24	40	0.52		2.07	(1.46–2.86)
	Missing	49625	45.57	3274	42.22		0.87	(0.83–0.91)
Human chorionic gonadotropin	Normal	58384	53.61	4328	55.82	<0.001	Reference	
	Abnormal	820	0.75	122	1.57		2.01	(1.64–2.42)
	Missing	49701	45.64	3304	42.61		0.89	(0.85–0.93)
Alpha-fetoprotein	Normal	58406	53.63	4190	54.04	<0.001	Reference	
	Abnormal	1365	1.25	318	4.10		3.42	(2.85–3.67)
	Missing	49134	45.12	3246	41.86		0.92	(0.87–0.96)
Diabetes during the second trimester	No diabetes	103303	94.86	6992	90.17	<0.001	Reference	
	Gestational diabetes	4932	4.53	524	6.76		1.57	(1.42–1.72)
	Type I	269	0.25	123	1.59		6.75	(5.43–8.35)
	Type II	376	0.35	108	1.39		4.24	(3.40–5.24)
	Type Unknown	25	0.02	7	0.09		4.13	(1.65–9.13)
Hypertensive disorder	None	95411	87.61	6080	78.41	<0.001	Reference	
	Gestational hypertension	4812	4.42	562	7.25		1.83	(1.67–2.01)
	Eclampsia	42	0.04	24	0.31		8.96	(5.35–14.68)
	HELLP	81	0.07	112	1.44		21.69	(16.31–28.99)
	Preeclampsia	654	0.60	288	3.71		6.91	(5.99–7.94)
	Unknown	7905	7.26	688	8.87		1.39	(1.25–1.48)
Infection(s)	No	79027	72.57	6055	78.09	<0.001	Reference	
	Yes	29878	27.43	1699	21.91		1.34	(1.27–1.42)
Medication exposure	No	20814	19.11	1444	18.62	<0.001	Reference	
	Vitamins and herbals	56399	51.79	3311	42.70		0.84	(0.79–0.90)
	Other medication	31692	29.10	2999	38.68		1.36	(1.27–1.45)
Sex of baby	Female	53141	48.80	3365	43.40	<0.001	Reference	
	Male	55764	51.20	4389	56.60		1.24	(1.18–1.30)
Complications during pregnancy	No complications	93777	86.11	2974	38.35	<0.001	Reference	
	Mild-moderate complications	4538	4.17	283	3.65		1.96	(1.73–2.22)
	Severe complications	10590	9.72	4497	58.00		13.39	(12.73–17.08)

IVF: *In vitro* fertilization; SD: standard deviation; Pre-existing maternal health conditions shown in S2 Table. Pre-existing mental health conditions shown in S3 Table.

#### Multivariable analysis

**Fig 1:**
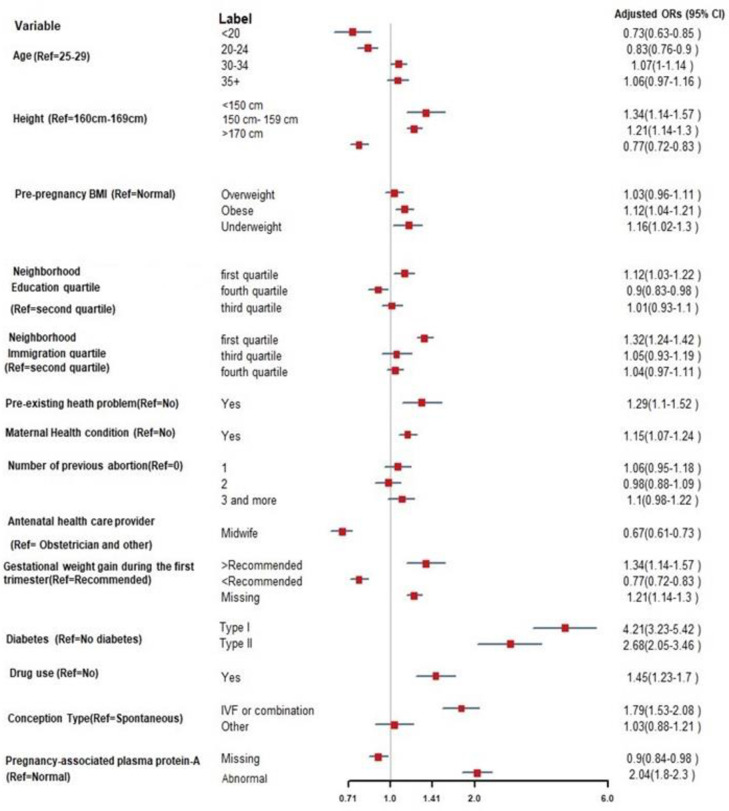
Selected variables and adjusted odds ratios during the first trimester for prediction of preterm birth in nulliparous women. BMI: Body mass index; IVF: *In vitro* fertilization; Ref: Reference group; Pre-existing maternal health conditions shown in S2 Table. Pre-existing mental health conditions shown in S3 Table. Number of previous abortions: includes the number of miscarriages.

Stepwise logistic regression identified 13 significant predictors during the first trimester (Fig 1). Diabetes (Type I: adjusted odds ratio (AOR): 4.21; 95% confidence interval (CI): 3.23–5.42; Type II: AOR: 2.68; 95% CI: 2.05–3.46) and abnormal pregnancy-associated plasma protein A concentrations (AOR: 2.04; 95% CI: 1.80–2.30) were the most significant predictors of PTB. The following factors were also associated with an increased risk of PTB: pregnancies conceived through IVF, being obese or underweight, maternal drug (substance) use, lower neighbourhood education level, lower neighbourhood immigration level, low maternal height, diabetes, and other pre-existing medical or mental health conditions.

**Fig 2:**
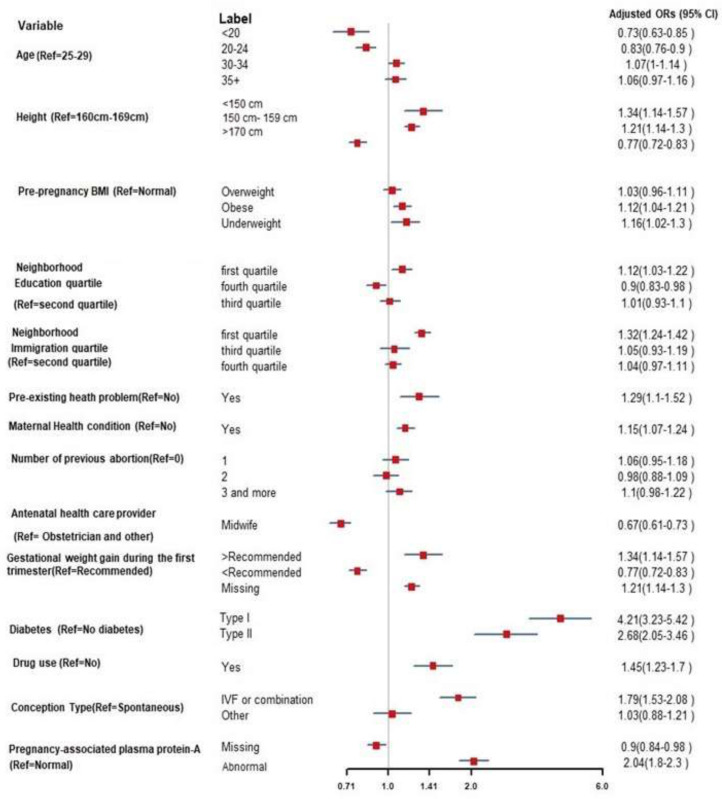
Selected variables and odds ratios during the second trimester for prediction of preterm birth in nulliparous women. BMI: Body mass index; IVF: *In vitro* fertilization; Ref: Reference group; Pre-existing maternal health conditions shown in S2 Table. Pre-existing mental health conditions shown in S3 Table. Number of previous abortions: includes the number of miscarriages.

During the second trimester, we identified 17 significant predictors related to PTB (Fig 2) using stepwise logistic regression. Many of the selected variables were the same as those selected for the first-trimester model, with slight changes in the odds ratios. Furthermore, severe complications of pregnancy were strongly associated with PTB (AOR: 13.03; 95% CI: 12.21–13.90). Women with abnormal alpha-fetoprotein, those carrying a male fetus, and those who did not attend prenatal classes were at increased odds of PTB. Exposure to medication during pregnancy, including vitamins and herbal supplements, was associated with a decreased risk of PTB.

Machine learning (Boruta) identified 17 and 27 important predictors of PTB during the first and second trimesters, respectively (S5 and S6 Tables). Unlike with logistic regression, machine learning models selected previous abortions (including miscarriages) as the most important predictor of PTB during the first trimester (importance: 28.23 for previous abortions (including miscarriages) vs. 7.79 for diabetes). During the second trimester, complications during pregnancy and hypertensive disorders were the most important predictors of PTB.

#### Prediction models and performance measures in the training and validation samples

**Fig 3:**
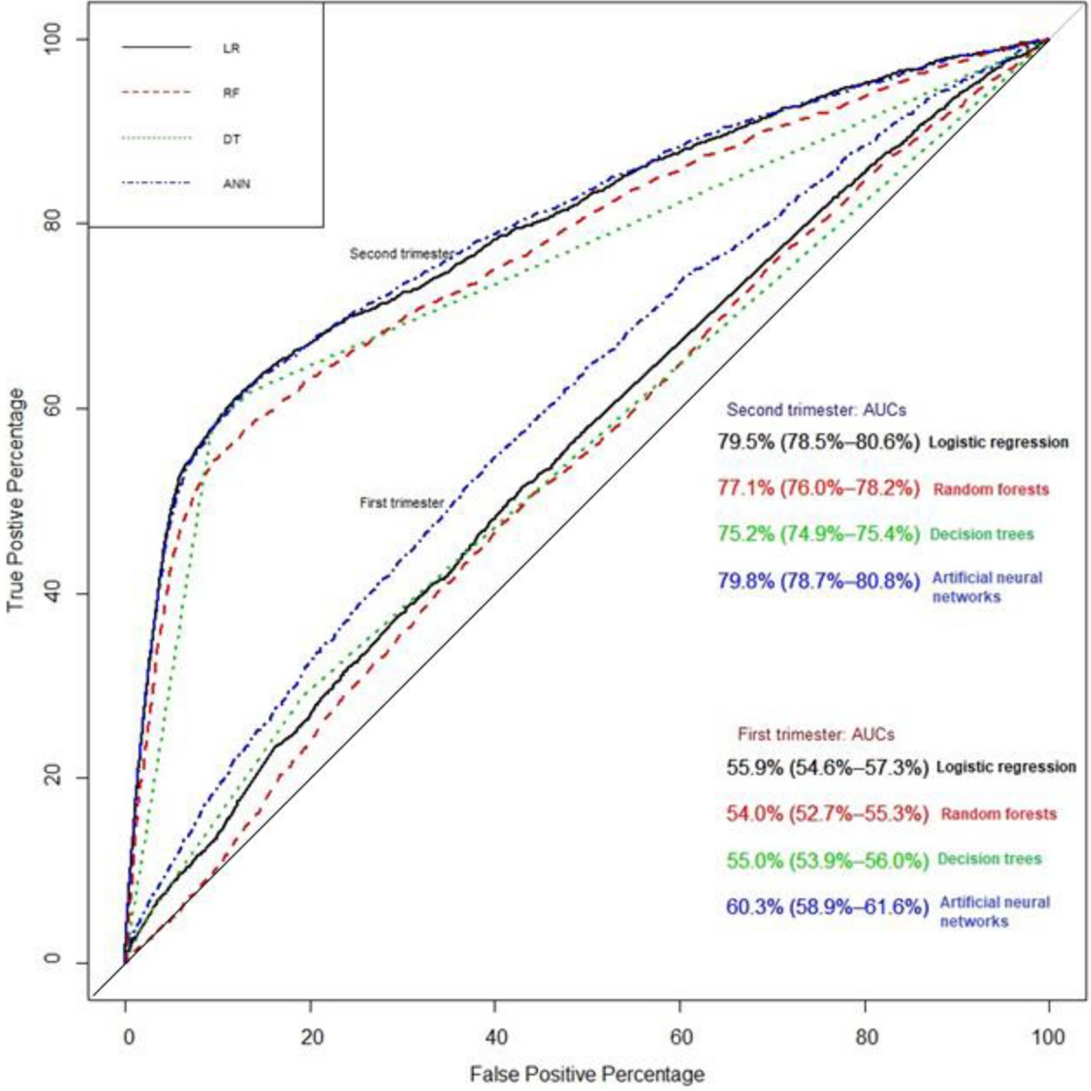
Comparison of prediction models during the first and second trimester for preterm birth in nulliparous women.

In the training sample, we found that random forests had a higher AUC than other models (99%), including logistic regression, which had the third highest AUC (S7 Table). We evaluated the proposed prediction models in the testing sample and found that during the first trimester the AUCs ranged from 53% (random forests) to 60% (artificial neural networks, Fig 3 and Table 4). However, all models had very high negative predictive values of ~95%. During the second trimester, artificial neural networks had the highest sensitivity of 63% (95% CI: 61–65%, Fig 3 and Table 4), but slightly lower specificity and positive predictive value than logistic regression. Random forests exhibited the lowest sensitivity among the models; however, the positive predictive value of the random forests model was the highest, but still relatively low at 36%.

**Table 4:** Predictive power of preterm birth models during the first and second trimesters in nulliparous women.

	First trimester				Second trimester			
Metric	Logistic regression	Random forests	Artificial neural networks	Decision trees	Logistic regression	Random forests	Artificial neural networks	Decision trees
Sensitivity	50.2 (47.8–52.4)	29.4 (26.1–31.6)	36.0 (34.5–42.3)	29.2 (27.1–30.8)	62.2 (60.0–63.4)	45.2 (44.5–48.5)	62.7 (61.2–65.4)	58.1 (55.6–60.2)
Specificity	64.5 (63.1–65.4)	84.5 (83.0–86.4)	71.2 (68.2–73.1)	80.2 (79.5–81.4)	87.0 (85.5–88.4)	94.1 (93.8–95.2)	84.6 (83.1–86.5)	90.1 (89.2–91.4)
Positive predictive value	8.5 (8.1–9.3)	11.4 (9.1–12.2)	11.3 (8.3–13.4)	9.2 (8.5–10.4)	25.2 (24.5–26.3)	36.0 (35.3–38.4)	23.2 (21.3–23).3	29.1 (27.1–29.2)
Negative predictive value	95.5 (94.4–95.3)	95.2 (94.9–96.1)	95.0 (94.1–95.3)	94.2 (93.9–95.2)	97.3 (96.3–98.3)	96.2 (95.6–97.2)	97.0 (96.5–98.2)	97.2 (96.1–98.4)

All values of percentages; 95% confidence intervals are given in parentheses.

Overall, there was an increase in the AUC from the first trimester to the second trimester in logistic regression and artificial neural networks (60% vs. 80%). The notable improvement of the AUC to 80% with artificial neural networks and logistic regression was due to the addition of complications during pregnancy (S1 and S3 Figs). All models provided negative predictive value of ~97% during the second trimester. In a sensitivity analysis, we compared the predictive power of all models without complications during pregnancy, and found that the AUC ranged from 58% (decision trees) to 65% (artificial neural networks, S1 Fig).

### Prediction of spontaneous PTB

For models predicting spontaneous PTB, during the first trimester the AUC ranged from 55% (random forests) to 59% (logistic regression, S2 Fig). During the second trimester, AUC ranged from 58% (decision trees) to 64% (logistic regression, S3 Fig). Both machine learning and logistic regression generated negative predictive values of approximately 94% for spontaneous PTB during the first and second trimesters (S8 Table). We emphasize that pregnancy complications, hypertensive disorder, and other medically induced PTB were not included in these analyses.

## Discussion

We used population-based data to predict PTB in nulliparous women using logistic regression and machine learning approaches during the first and second trimesters. We found that diabetes mellitus, a history of spontaneous or therapeutic abortions, and abnormal pregnancy-associated plasma protein A concentrations were the strongest predictors for PTB during the first trimester. Thirteen selected predictors yielded a maximum AUC of 60% with artificial neural networks, thus providing poor prediction of PTB during the first trimester, even using machine learning approaches. During the second trimester, 17 variables were significantly associated with PTB, among which complications during pregnancy had the highest AOR (13.03; 95% CI: 12.21–13.9). During the second trimester, the AUC increased from 65% (95% CI: 63–66%) to 80% (95% CI: 79–81%) with the inclusion of complications during pregnancy, which is a moderate predictor [50] of PTB.

Machine learning identified more variables associated with PTB than logistic regression in our data set. During the first trimester, machine learning identified previous abortions (which includes miscarriages) as the strongest predictor of PTB, while logistic regression identified diabetes as the strongest predictor. A history of prior abortions (including miscarriages) may be a more important predictor of PTB because the incidence of prior abortions was substantially higher than that of diabetes.

We found that conventional logistic regression and machine learning had comparable performance for prediction of PTB. Other studies comparing machine learning methods to conventional logistic regression for the prediction of a variety of clinical conditions showed that in general, no single method consistently provided the best prediction [51–58]. Although logistic regression is a frequently used method, it requires linearity and independence between the predictors. Conversely, machine learning is a non-parametric approach that can handle complex and non-linear models.

There was a significant decrease in the AUC between the training and the testing data, possibly due to the overfitting problem of machine learning methods [54]. Specifically, random forests are “greedy”, and thus, try to minimize the error in the training sample, which may cause overfitting (high performance in training but lower performance in the validation sample, as we observed in our models) [30].

Accurate prediction of PTB in nulliparous women has been lacking. Woolery and Grzymala [55] found machine learning had 53–88% accuracy in predicting PTB. Using data mining methods, Goodwin *et al*. found that seven demographic variables produced an AUC of 72% [10]. In contrast, Grobman *et al*. [12] found that logistic regression provided poor performance (AUC, 63%) for prediction of PTB in nulliparous women with a short cervix. Catley *et al*. [15] explored artificial neural networks for the prediction of PTB in high-risk pregnant women and found model sensitivity of 20% before 22 weeks of gestation. Weber *et al*. [13] recently applied machine learning to predict early (<32 weeks) spontaneous PTB among nulliparous women and found an AUC of only 63–65%, similar to Courtney *et al*. [56] (AUC, 60%) using logistic regression and a support vector machine approach.

### Strengths of the study

Our study had several strengths. Firstly, our models generated high negative predictive values, higher than fetal fibronectin for spontaneous PTB [57], and thus may lead to reduction in unnecessary resource use [58]. Secondly, we considered a wide range of variables available in standard clinical care databases (e.g., proteins for screening for Down syndrome or placental diseases, gestational weight gain) that were not considered in previous studies. Another strength of the current work is the consideration of different time points (first and second trimesters) for the prediction of PTB. In addition, we evaluated a relatively large cohort, particularly compared to many of the previous studies [8–14]. We considered multiple methods for variable selection and prediction to maximize accuracy. We addressed several limitations of previous studies in this area: Courtney *et al*. [56] found that logistic regression and machine learning models based on demographic data were not able to predict PTB adequately (AUC, 60%). Those authors suggested that prenatal demographic factors such as maternal health behaviors and medical history could be used to construct accurate models, and thus, we included such factors in our study. By performing a large cohort study, we also addressed the “lack of data” problem identified in the work of Lee *et al*. [11]. We applied multiple imputation (repeated ten times), which is a robust technique for handling missing data [48]. Unlike Fergue *et al*. [16], we used random oversampling in the training set only, thus the AUC from our models was generated from clinical data and not artificial samples.

### Limitations

Our study also has several limitations, including the low predictive power of the proposed models, particularly during the first trimester. The predictive ability of all models strongly depends on the predictor variables [30]. Although we had a large number of variables and a relatively large number of subjects, one of the limitations of our prediction models was the lack of information on the interventions used for pregnancies at high risk of PTB. However, data suggest relatively low rates of use of such preventive measures in our study population [59]. We categorized PTB as <37 or ≥37 weeks of gestation, which may lead to loss of statistical power [60]. Further, binary categorization collapses all types of PTB in one group despite different rates of neonatal mortality and morbidity for each category of PTB [61] and despite potentially different predictors of extremely PTB compared to PTB overall. Although low pregnancy-associated plasma protein A concentraion is associated with trisomies which themselves are associated with preterm birth, the majority of such cases are in euploid pregnancies [62–66]. Finally, we were unable to examine ultrasonographic measurement of the uterine cervix, which is a strong predictor of PTB [67] as it is not available in the BORN database.

## Conclusion

Including data from the second trimester improved prediction power to a moderate level of 80% AUC by both logistic regression and machine learning. However, developing an accurate prediction model during the first trimester will require further investigation. Inclusion of data from additional biomarkers may increase prediction accuracy.

## Supporting information

S1 Fig Receiver operating characteristic curves for second-trimester prediction models without the “complications during pregnancy” variable in the validation sample. (DOCX)

S2 Fig Receiver operating characteristic curves for first-trimester prediction models for spontaneous preterm birth in the validation sample. (DOCX)

S3 Fig Receiver operating characteristic curves for second-trimester prediction models for spontaneous preterm birth in the validation sample. (DOCX)

S1 Table Definitions of neighbourhood income, immigration, education, and minority quartiles. (DOCX)

S2 Table Pre-existing maternal health conditions. (DOCX)

S3 Table Pre-existing mental health conditions. (DOCX)

S4 Table Cut-off points for nuchal translucency and protein concentrations. (DOCX)

S5 Table Variables selected by the machine learning algorithm for prediction of preterm birth during the first trimester in nulliparous women. (DOCX)

S6 Table Variables selected by the machine learning algorithm for prediction of preterm birth during the second trimester in nulliparous women. (DOCX)

S7 Table Optimal hyperparameters, sensitivity, specificity, and area under the receiver operating characteristic curve in training samples. (DOCX)

S8 Table Predictive power of spontaneous preterm birth models during the first and second trimesters in the testing data. (DOCX)
