# Supplementary material for: Prediction of preterm birth in nulliparous women using logistic regression and machine learning
Source: PLoS One. 2021 Jun 30;16(6):e0252025. doi: 10.1371/journal.pone.0252025 (PMC8244906; doi:10.1371/journal.pone.0252025)
Supplement: S2 Fig — (DOCX) [file pone.0252025.s002.docx]

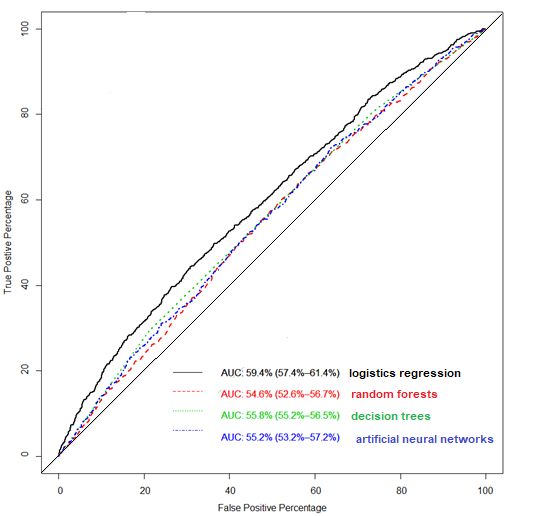


S2 Fig: Receiver operating characteristic curves for first-trimester prediction models for spontaneous preterm birth in the validation sample.
