# Supplementary material for: Prediction of preterm birth in nulliparous women using logistic regression and machine learning
Source: PLoS One. 2021 Jun 30;16(6):e0252025. doi: 10.1371/journal.pone.0252025 (PMC8244906; doi:10.1371/journal.pone.0252025)
Supplement: S1 Table — (DOCX) [file pone.0252025.s004.docx]

S1 Table: Definitions of neighbourhood income, immigration, education, and minority quartiles

| Neighbourhood income quartile | Neighbourhood median household income level represents the median household income of the total population residing in a given neighbourhood. |
| --- | --- |
|  | Neighbourhood median household income values distributed by dissemination area were categorized by quartile. Each quartile represents approximately a quarter of the total population of Ontario but does not necessarily represent a quarter of births in Ontario. |
| Neighbourhood immigration quartile | The neighbourhood immigrant concentration represents the number of immigrants in a neighbourhood, expressed as a percentage of the total population of that neighbourhood. |
|  | Lowest quartile values for immigrant status represent the lower portion of the distribution by dissemination area of immigrants in a neighbourhood relative to the highest quartile values, which represent the upper portion of the distribution of immigrants. |
|  | Neighbourhood immigrant status values distributed by dissemination area were categorized by quartile. Each quartile represents approximately a quarter of the total population of Ontario but does not necessarily represent a quarter of births in Ontario. |
| Neighbourhood education quartile | The neighbourhood education level status represents the number of people in a neighbourhood who do not hold a diploma, expressed as a percentage of the total population aged 25 to 64 years within that neighbourhood. |
|  | Lowest quartile values for neighbourhood education level represent the lower portion of the distribution by dissemination area of individuals who have not completed a certificate, degree, or diploma in a neighbourhood relative to highest quartile values, which represent the upper portion of the distribution who have NOT completed a certificate, degree, or diploma. |
|  | Neighbourhood education level values distributed by dissemination area were categorized by quartile. Each quartile represents approximately a quarter of the total population of Ontario but does not necessarily represent a quarter of births in Ontario. |
| Neighbourhood minority quartile | Visible minority is defined in the 2011 Statistics Canada Long-Form Census as persons, other than Aboriginal peoples, who are non-Caucasian in race or non-white in colour. |
|  | The neighbourhood visible minority status represents the number of people in a neighbourhood who self-identified as belonging to a visible minority, expressed as a percentage of the total population in that neighbourhood. |
|  | Neighbourhood visible minority status values distributed by dissemination area were categorized by quartile. Each quartile represents approximately a quarter of the total population of Ontario but does not necessarily represent a quarter of births in Ontario. |
