# Supplementary material for: Prediction of preterm birth in nulliparous women using logistic regression and machine learning
Source: PLoS One. 2021 Jun 30;16(6):e0252025. doi: 10.1371/journal.pone.0252025 (PMC8244906; doi:10.1371/journal.pone.0252025)
Supplement: S2 Table — (DOCX) [file pone.0252025.s005.docx]

S2 Table: Pre-existing maternal health conditions

| Autoimmune |
| --- |
| Autoimmune / Lupus |
| Autoimmune / Other |
| Autoimmune / Rheumatoid Arthritis |
| Cancer |
| Cancer / Diagnosed in Pregnancy |
| Cancer / Prior to Pregnancy |
| Cardiovascular |
| Cardiovascular / Acquired Heart Disease |
| Cardiovascular / Antihypertensive Therapy outside of pregnancy |
| Cardiovascular / Cardiovascular Disease |
| Cardiovascular / Congenital Heart Defect |
| Cardiovascular / Congenital Heart Disease |
| Cardiovascular / Other |
| Cardiovascular / Pre-existing Hypertension |
| Cardiovascular / Renal Disease |
| Craniofacial |
| Craniofacial / Cleft Lip and/or palate |
| Craniofacial / Craniosynostosis |
| Craniofacial / Other |
| Diabetes |
| Diabetes / Diabetes Type I |
| Diabetes / Diabetes Type II - Insulin |
| Diabetes / Diabetes Type II - No Insulin |
| Diabetes / Diabetes Type II - No Insulin / Diet management only |
| Diabetes / Diabetes Type II - No Insulin / Oral Antihyperglycemic Agents |
| Diabetes / Diabetes Type Unknown |
| Endocrine |
| Endocrine / Hyperthyroidism \| Managed |
| Endocrine / Hyperthyroidism \| Management Unknown |
| Endocrine / Hyperthyroidism \| Unmanaged |
| Endocrine / Hypothyroidism \| Managed |
| Endocrine / Hypothyroidism \| Management Unknown |
| Endocrine / Hypothyroidism \| Unmanaged |
| Endocrine / Other |
| Endocrine / Thyroid disease |
| Gastrointestinal |
| Gastrointestinal / Colitis |
| Gastrointestinal / Crohn’s |
| Gastrointestinal / Hepatitis |
| Gastrointestinal / Liver |
| Gastrointestinal / Liver/Gallbladder \| Cholecystitis |
| Gastrointestinal / Liver/Gallbladder \| Intrahepatic cholestasis of pregnancy |
| Gastrointestinal / Liver/Gallbladder \| Other |
| Gastrointestinal / Other |
| Genetics |
| Genetics / CGH Microarray abnormality polymorphism |
| Genetics / Chromosome Abnormality |
| Genetics / Chromosome Rearrangement (balanced) |
| Genetics / Other birth defects |
| Genetics / Other genetic inherited disorders/syndromes |

S2 Table: Pre-existing maternal health conditions (Continued)

| Genito Urinary |
| --- |
| Genito Urinary / Acquired Renal (insufficiency - chronic infections) |
| Genito Urinary / Congenital/Genetic Renal (renal agenesis - pelvic kidney) |
| Genito Urinary / Other |
| Genito Urinary / Renal Disease |
| Genito Urinary / Uterine Anomalies |
| Haematology |
| Haematology / Chronic Anemia |
| Haematology / Gestational Thrombocytopenia |
| Haematology / Haemophilia (A and B von Willebrand) |
| Haematology / Idiopathic Thrombocytopenia |
| Haematology / Other |
| Haematology / Sickle Cell Disease |
| Haematology / Thalassemia |
| Haematology / Thrombophilia |
| Infection |
| Musculoskeletal |
| Musculoskeletal / Achondroplasia |
| Musculoskeletal / Limb Reduction Defects |
| Musculoskeletal / Muscular Dystrophy/Neuromuscular disorder |
| Musculoskeletal / Musculoskeletal (Unspecified) |
| Musculoskeletal / Myotonic Dystrophy |
| Musculoskeletal / Osteogenesis Imperfecta |
| Musculoskeletal / Other |
| Musculoskeletal / Unspecified |
| Neurodevelopmental |
| Neurodevelopmental / Autism |
| Neurodevelopmental / Developmental Delay (Mental Retardation) |
| Neurodevelopmental / Fragile X |
| Neurodevelopmental / Hearing Disorder |
| Neurodevelopmental / Learning Disabilities |
| Neurodevelopmental / Other |
| Neurodevelopmental / Vision Disorder |
| Neurology |
| Neurology / Cerebral palsy |
| Neurology / Epilepsy/Seizures \| Pre-existing |
| Neurology / Epilepsy/Seizures \| Seizure occurred in current pregnancy |
| Neurology / Multiple Sclerosis |
| Neurology / Myasthenia Gravis |
| Neurology / Other |
| Neurology / Spina Bifida/NTD |
| Other, specify: |
| Pulmonary |
| Pulmonary / Asthma \| Occurred in current pregnancy |
| Pulmonary / Asthma \| Pre-existing |
| Pulmonary / Cystic Fibrosis |
| Pulmonary / Other |
| Pulmonary / Previous Pulmonary Embolism/DVT |
| Pulmonary / Pulmonary Hypertension |
| Recurrent Spontaneous Abortion |
| Unknown |
