# Supplementary material for: Prediction of preterm birth in nulliparous women using logistic regression and machine learning
Source: PLoS One. 2021 Jun 30;16(6):e0252025. doi: 10.1371/journal.pone.0252025 (PMC8244906; doi:10.1371/journal.pone.0252025)
Supplement: S3 Table — (DOCX) [file pone.0252025.s006.docx]

S3 Table: Pre-existing mental health conditions

| Addiction |
| --- |
| Anxiety |
| Bipolar Disorder |
| Depression |
| History of Postpartum Depression |
| Other |
| Schizophrenia |
| Unknown |
