# Supplementary material for: Prediction of preterm birth in nulliparous women using logistic regression and machine learning
Source: PLoS One. 2021 Jun 30;16(6):e0252025. doi: 10.1371/journal.pone.0252025 (PMC8244906; doi:10.1371/journal.pone.0252025)
Supplement: S4 Table — (DOCX) [file pone.0252025.s007.docx]

S4 Table: Cut-off points for nuchal translucency and protein concentrations

|  | Measurements | Normal range | Abnormal range |  |
| --- | --- | --- | --- | --- |
| First trimester | Nuchal translucency level | <3.5 mm | ≥3.5 mm |  |
|  | **Proteins** | | | |
|  | Multiples of the median for pregnancy-associated plasma protein A | >0.4 | ≤0.4 |  |
|  | Multiples of the median for free beta-subunit of human chorionic gonadotropin | <2.31 | ≥2.31 |  |
| Second trimester | Multiples of the median for dimeric inhibin A | <2 | ≥2 |  |
|  | Multiples of the median for human chorionic gonadotropin | <3 | ≥3 |  |
|  | Multiples of the median for unconjugated estriol | <2 | ≥2 |  |
|  | Multiples of the median for alpha-fetoprotein | <2 | ≥2 |  |

Cut-off points for nuchal translucency and proteins are based on our literature search and on guidelines from the American College of Obstetricians and Gynecologists and the International Society of Ultrasound in Obstetrics and Gynecology.
