# Supplementary material for: Prediction of preterm birth in nulliparous women using logistic regression and machine learning
Source: PLoS One. 2021 Jun 30;16(6):e0252025. doi: 10.1371/journal.pone.0252025 (PMC8244906; doi:10.1371/journal.pone.0252025)
Supplement: S5 Table — (DOCX) [file pone.0252025.s008.docx]

S5 Table: Variables selected by the machine learning algorithm for prediction of preterm birth during the first trimester in nulliparous women

| Variable | Mean importance | Median importance | Minimum importance | Maximum importance |
| --- | --- | --- | --- | --- |
| Number of previous abortions | 28.23 | 28.39 | 24.23 | 32.74 |
| Neighbourhood minority quartile | 27.91 | 27.80 | 23.54 | 31.98 |
| Neighborhood immigration quartile | 25.74 | 25.97 | 20.74 | 30.34 |
| Pregnancy-associated plasma protein A | 23.57 | 23.75 | 18.72 | 27.81 |
| Weight gain during the first trimester | 19.98 | 19.94 | 15.26 | 24.50 |
| Smoking status | 15.61 | 15.63 | 12.45 | 19.09 |
| Age | 15.09 | 15.16 | 11.02 | 18.65 |
| Ex-smoker | 14.62 | 14.70 | 11.24 | 19.79 |
| Conception type | 11.91 | 11.79 | 6.73 | 15.35 |
| Folic acid use | 9.53 | 9.62 | 5.89 | 12.45 |
| Diabetes during the first trimester | 7.79 | 7.71 | 3.54 | 11.68 |
| Pre-existing health conditions | 7.07 | 6.96 | 4.30 | 9.64 |
| Drug (substance) use | 6.85 | 6.68 | 4.22 | 10.49 |
| Neighbourhood education quartile | 6.74 | 6.82 | 3.24 | 9.95 |
| Neighbourhood income quartile | 5.91 | 5.90 | 2.29 | 9.65 |
| Antenatal health care provider | 3.51 | 3.41 | -0.18 | 7.74 |
| Pre-existing mental health conditions | 2.43 | 2.41 | -0.47 | 5.92 |
