# Supplementary material for: Prediction of preterm birth in nulliparous women using logistic regression and machine learning
Source: PLoS One. 2021 Jun 30;16(6):e0252025. doi: 10.1371/journal.pone.0252025 (PMC8244906; doi:10.1371/journal.pone.0252025)
Supplement: S6 Table — (DOCX) [file pone.0252025.s009.docx]

S6 Table: Variables selected by the machine learning algorithm for prediction of preterm birth during the second trimester in nulliparous women

| Variable | Mean importance | Median importance | Minimum importance | Maximum importance |
| --- | --- | --- | --- | --- |
| Complications during pregnancy | 144.35 | 145.55 | 131.87 | 156.86 |
| Hypertensive disorder | 72.51 | 72.24 | 66.37 | 79.37 |
| Diabetes during the first trimester | 22.51 | 22.49 | 19.78 | 24.58 |
| Pregnancy-associated plasma protein A | 21.19 | 21.43 | 17.28 | 24.73 |
| Alpha-fetoprotein | 20.63 | 20.56 | 16.90 | 23.26 |
| First trimester visit | 20.56 | 20.57 | 18.17 | 23.50 |
| Neighborhood immigration quartile | 20.10 | 20.01 | 17.33 | 23.32 |
| Neighborhood minority quartile | 18.24 | 18.17 | 15.76 | 21.39 |
| Prenatal classes | 16.10 | 16.10 | 13.07 | 19.23 |
| Age | 15.73 | 15.68 | 12.78 | 19.01 |
| Weight gain during the first trimester | 14.72 | 14.91 | 12.16 | 16.69 |
| Number of previous abortions | 12.87 | 12.83 | 6.64 | 20.21 |
| Smoking status | 11.75 | 11.72 | 8.56 | 14.72 |
| Antenatal health care provider | 10.89 | 10.74 | 8.75 | 13.41 |
| Ex-smoker | 10.50 | 10.70 | 6.87 | 13.08 |
| Drug (substance) use | 9.38 | 9.55 | 6.89 | 11.75 |
| Conception type | 7.98 | 8.04 | 5.59 | 9.96 |
| Free beta-subunit of human chorionic gonadotropin | 7.54 | 7.36 | 5.44 | 9.69 |
| Pre-existing health conditions | 7.11 | 7.21 | 4.24 | 9.08 |
| Pre-pregnancy body mass index | 6.61 | 6.51 | 4.24 | 9.48 |
| Folic acid use | 6.11 | 6.14 | 3.67 | 8.48 |
| Neighborhood education quartile | 5.78 | 5.87 | 3.62 | 8.11 |
| Medication exposure | 5.25 | 5.31 | 2.89 | 7.36 |
| Dimeric inhibin A | 4.05 | 3.95 | 1.89 | 6.13 |
| Pre-existing mental health conditions | 3.51 | 3.75 | 1.46 | 5.56 |
| Height | 3.31 | 3.30 | 1.06 | 5.27 |
| Neighbourhood income quartile | 3.10 | 3.15 | 1.33 | 5.33 |
