# Supplementary material for: Prediction of preterm birth in nulliparous women using logistic regression and machine learning
Source: PLoS One. 2021 Jun 30;16(6):e0252025. doi: 10.1371/journal.pone.0252025 (PMC8244906; doi:10.1371/journal.pone.0252025)
Supplement: S7 Table — (DOCX) [file pone.0252025.s010.docx]

S7 Table: Optimal hyperparameters, sensitivity, specificity, and area under the receiver operating characteristic curve in training samples

| First trimester | | | | |
| --- | --- | --- | --- | --- |
| Method | Optimal hyperparameters in training sample ^1^ | Sensitivity | Specificity | Area under the receiver operating characteristic curve |
| Logistic regression | - | 56.2% | 62.7% | 60.3% |
| Decision trees | ^2^Complexity parameter (cp) = 0.008 | 57.4% | 62.3% | 61.8% |
| Random forests | ^3^mtry = 24 | 98.1% | 92.4% | 97.6% |
| Artificial neural networks | ^4^Size = 5; decay = 0.0001 | 57.4% | 64.8% | 64.4% |
| Second trimester | | | | |
| Method | Optimal hyperparameters in training sample ^1^ | Sensitivity | Specificity | Area under the receiver operating characteristic curve |
| Logistic regression | - | 63.2% | 83.3% | 80.6% |
| Decision trees | ^2^Complexity parameter (cp) = 0.002 | 60.4% | 90.1% | 76.5% |
| Random forests | ^3^mtry = 35 | 99.1% | 99.3% | 95.7% |
| Artificial neural networks | ^4^Size = 5; decay = 0.1 | 64.6% | 86.1% | 82.2% |

^1^The area under the receiver operating characteristic curve (maximum) was used to select the optimal model. ^2^The complexity parameter (cp) is used to control the size of the decision tree and to select the optimal tree size. If the cost of adding an additional variable to the decision tree from the current node is above the value of the cp, then tree building does not continue. ^3^mtry is the number of variables available for splitting at each tree node. In the random forests literature, this is referred to as the mtry parameter. ^4^Size is the number of units in a hidden layer. Decay is the regularization parameter used to avoid over-fitting.
