# Supplementary material for: Prediction of preterm birth in nulliparous women using logistic regression and machine learning
Source: PLoS One. 2021 Jun 30;16(6):e0252025. doi: 10.1371/journal.pone.0252025 (PMC8244906; doi:10.1371/journal.pone.0252025)
Supplement: S8 Table — (DOCX) [file pone.0252025.s011.docx]

S8 Table: Predictive power of spontaneous preterm birth models during the first and second trimesters in the testing data

|  | First trimester | | | | Second trimester | | | |
| --- | --- | --- | --- | --- | --- | --- | --- | --- |
| Metric | Logistic regression | Random forests | Artificial neural networks | Decision trees | Logistic regression | Random forests | Artificial neural networks | Decision trees |
| Sensitivity | 52.5 (49.1-55.9) | 52.3 (48.9-55.7) | 76.1 (73.2-79.0) | 66.3 (63.1-69.5) | 57.4 (54.5-60.2) | 54.9 (52.1-57.8) | 49.7 (46.9-52.6) | 52.9 (50.0-55.7) |
| Specificity | 61.1 (60.3-62.0) | 61.9 (61.0-62.8) | 27.5 (26.7-28.3) | 41.2 (40.3-42.1) | 65.0 (64.3-65.8) | 66.1 (65.4-66.9) | 64.4 (63.6-65.1) | 62.6 (61.8-63.4) |
| Positive predictive value | 8.5 (7.7-9.3) | 8.6 (7.8-9.4) | 6.7 (6.2-7.2) | 7.2 (6.6-7.8) | 11.0 (10.2-11.7) | 10.9 (10.1-11.7) | 9.5 (8.7-10.2) | 9.6 (8.9-10.3) |
| Negative predictive value | 94.9 (94.5-95.4) | 95.0 (94.5-95.5) | 94.4 (93.6-95.1) | 94.7 (94.1-95.3) | 95.3 (94.9-95.7) | 95.1 (94.7-95.5) | 94.5 (94.0-94.9) | 94.7 (94.2-95.1) |

All values of percentages; 95% confidence intervals are given in parentheses.
