# Supplementary material for: Prediction of preterm birth in nulliparous women using logistic regression and machine learning
Source: PLoS One. 2021 Jun 30;16(6):e0252025. doi: 10.1371/journal.pone.0252025 (PMC8244906; doi:10.1371/journal.pone.0252025)
Supplement: Attachment — Submitted filename: Response to the Reviwers comments_R1 GS.docx [file pone.0252025.s012.docx]

Dear Editor and Reviewers,

We greatly appreciate your careful reading of our manuscript and the helpful feedback you have provided. We have revised the manuscript based on your comments, as detailed below. We thank you again for your valuable time and we hope that you find our revised manuscript acceptable for publication. Our responses to your comments are given in Blue font. We also added the requested revisions in the text in the Blue font. We also reformatted the paper according to the journal guidelines.

**Comments to the Author**

1. Is the manuscript technically sound, and do the data support the conclusions?

The manuscript must describe a technically sound piece of scientific research with data that supports the conclusions. Experiments must have been conducted rigorously, with appropriate controls, replication, and sample sizes. The conclusions must be drawn appropriately based on the data presented.

Reviewer #1: Yes

Reviewer #2: Yes

2. Has the statistical analysis been performed appropriately and rigorously?

Reviewer #1: Yes

Reviewer #2: Yes

3. Have the authors made all data underlying the findings in their manuscript fully available?

The [PLOS Data policy](http://www.plosone.org/static/policies.action#sharing) requires authors to make all data underlying the findings described in their manuscript fully available without restriction, with rare exception (please refer to the Data Availability Statement in the manuscript PDF file). The data should be provided as part of the manuscript or its supporting information, or deposited to a public repository. For example, in addition to summary statistics, the data points behind means, medians and variance measures should be available. If there are restrictions on publicly sharing data—e.g. participant privacy or use of data from a third party—those must be specified.

Reviewer #1: Yes

Reviewer #2: Yes

4. Is the manuscript presented in an intelligible fashion and written in standard English?

PLOS ONE does not copyedit accepted manuscripts, so the language in submitted articles must be clear, correct, and unambiguous. Any typographical or grammatical errors should be corrected at revision, so please note any specific errors here.

Reviewer #1: No

Reviewer #2: Yes

5. Review Comments to the Author

Please use the space provided to explain your answers to the questions above. You may also include additional comments for the author, including concerns about dual publication, research ethics, or publication ethics. (Please upload your review as an attachment if it exceeds 20,000 characters)

**Reviewer #1: In this study Authors constructed by using logistic regression analysis and machine learning technique an algorithm to predict preterm labor defined as < 37 weeks. The argument is of interest, the number of women considered relevant and an elegant statistical approach was used. So I would like to congratulate with Authors for their effort
My comments are as follows**

1) did Authors differentiate spontaneous from iatrogenic preterm delivery? This is of crucial since women with pregestational diseases (diabetes) or developing medical complications are frequently induced preterm and this may flaw the algorithm

We thank the reviewer for this important comment. We did examine spontaneous PTB as a secondary outcome, excluding medically induced PTB and women with PPROM. Results from this analysis are presented in the last paragraph of the Results section and are included below for your convenience.

Prediction of spontaneous PTB

For models predicting spontaneous PTB, during the first trimester the AUC ranged from 55% (random forests) to 59% (logistic regression, S2 Fig). During the second trimester, AUC ranged from 58% (decision trees) to 64% (logistic regression, S3 Fig). Both machine learning and logistic regression generated negative predictive values of approximately 94% for spontaneous PTB during the first and second trimesters (Supplementary Table 8). We emphasize that pregnancy complications, hypertensive disorder, and other medically induced PTB were not included in these analyses.

2) Although stated as a limitation I suggest Authors to perform their analysis also at earlier gestational age (e.g. < 34 and or 32 weeks) that are more clinical significant

We previously developed separate prediction models for preterm birth <32 weeks and <28 weeks. Because of the differing prevalence, and risk factors, between these outcomes and PTB <37 weeks and because the analyses for the present manuscript are focused on nulliparous women, we chose to publish the models for earlier PTB outcomes in a separate manuscript, which is currently in press elsewhere.

3) It should be acknowledged that data on ultrasonographic measurement of the uterine cervix are missing since at present it is considered the powerful predictive variables.

We thank the reviewer for this suggestion. Although ultrasonographic measurement of the uterine cervix during the second trimester is indeed a strong predictor of preterm birth, the BORN database does not include data on such measurements. We now discuss this in the limitations section (last paragraph of the Discussion).

**Reviewer #2: In this manuscript, Belaghi et al use a database of nulliparous women who delivered in Ontario, Canada to predict PTB using both logistic regression and machine learning techniques. They found that using data available from the second trimester improved their prediction models using both approaches. The paper is well-written and easy to understand. However, several important questions arise from this study in its current form:**

1. Spontaneous PTB: How was this defined? This is not clear from the manuscript. This should be clarified.

We defined spontaneous PTB using the definition from Maghsouldu *et al.* (2019), as follows: not “induced”, not “caesarean section” and not “augmented labor”. We now clarify the definition of spontaneous PTB in the outcome subsection (second paragraph of the Methods).

Maghsoudlou, S., Yu, Z. M., Beyene, J., & McDonald, S. D. (2019). Phenotypic classification of preterm birth among nulliparous women: a population-based cohort study. *Journal of Obstetrics and Gynaecology Canada*, *41*(10), 1423-1432.

Further, as the authors allude but do not directly discuss, PTB can be broadly classified into provider-initiated PTB and spontaneous PTB. The pathophysiology of spontaneous PTB is very different than that of provider-initiated PTB. Although this study is by no means the first study to group PTB broadly into one category, it should directly address the reality that PTB has many phenotypes and that a prediction algorithm that is trying to predict all PTB inherently has many limitations. An algorithm that predicts spontaneous PTB may be of greater utility and greater accuracy than an algorithm that tries to predict both spontaneous PTB and HELLP syndrome necessitating provider-initiated delivery.

In addition to our principal analyses, we also developed prediction models for spontaneous PTB as a secondary outcome. The results from this analysis are reported in the last paragraph of the Results section.

Further, in the abstract, the authors compare their model to the negative predictive value of a fetal fibronectin test. A fetal fibronectin test is ONLY used to predict spontaneous PTB, not all PTB. Consequently, this comparison is of little utility.

In line with the reviewer’s suggestion, we have removed the comparison between our predictive model for overall PTB and FFN from the abstract, and we now refer to FFN only in connection with spontaneous PTB.

Further, what percentage of PTB included in this study was spontaneous? This is not clear from the manuscript. If possible, the authors should provider information on the various phenotypes of PTB and how they were ascertained. This information is of significant clinical utility.

There were a total of 3695 spontaneous preterm births in our analytic data set, accounting for 53% of all PTB (3695/6955) and yielding a spontaneous PTB rate of 5.62% (3695/65644). We now clarify this in the footnote to Table 1. We ascertained spontaneous PTB using the definition from Maghsouldu *et* *al.*, as discussed in our response to point 1 above and in the second paragraph of the Methods section.

Maghsoudlou, S., Yu, Z. M., Beyene, J., & McDonald, S. D. (2019). Phenotypic classification of preterm birth among nulliparous women: a population-based cohort study. *Journal of Obstetrics and Gynaecology Canada*, *41*(10), 1423-1432.

2. PAPP-A: In the abstract, the authors mention "abnormal pregnancy-associated plasma protein-A contractions" as being strongly associated with PTB. However, how a PAPP-A contraction was defined is unclear, as this contraction is never mentioned again. Is this meant to read concentration, not contraction?

We thank the reviewer for drawing our attention to this error. This word was indeed intended to be “concentration” and we have changed it accordingly.

3. Complications during pregnancy: No definition of moderate complications is provided in the manuscript. Additionally, what percentage of women had each of the severe complications listed on page 6 is not clear. The only clarity regarding this variable is provided on page 6: "The variable, complications during pregnancy, had more than 600 categories and we classified those data into three groups based on the expert opinion of our in-home maternal-fetal specialist, including no complications, moderate complications, and severe complications (including hypertensive disorder, placental abnormalities, and maternal complications during this pregnancy, such as antepartum bleeding)." As severe complications of pregnancy were highly associated with PTB, it would be helpful to better understand this variable. Further, if possible, these complications should be separated and included in the model, as one would expect preeclampsia and HELLP are more likely to lead to provider-initiated PTB and antepartum bleeding to be associated with abruption and preterm labor, which would likely lead to spontaneous PTB.

We thank the reviewer for bringing this point to our attention. Categorization of complications as mild-moderate complications versus severe was based on expert maternal-fetal input (Dr. Sarah McDonald). We now clarify this in the last paragraph of the Predictors subsection, just above Statistical Analysis.

The distribution of complications during pregnancy, which we have added to the end of Table 1, is as follows:

| Complications during pregnancy | N | Percent |
| --- | --- | --- |
| No complications | 90302 | 79.94 |
| Mild-Moderate complications | 4676 | 4.14 |
| Severe complications | 14255 | 12.62 |
| Missing | 3730 | 3.30 |

This variable was not included in the first-trimester prediction models for overall or spontaneous PTB, whereas it was included in the second-trimester prediction model for overall PTB but not for the spontaneous PTB. We report on the significant predictor variables included in the different models beginning in paragraph 4 of the Results.

We examined the predictive power of all models without complications during pregnancy as a sensitivity analysis, reported in the second to last paragraph of the Results. The AUC in models without complications during pregnancy ranged from 58% (decision trees) to 65% (artificial neural networks, S1 Fig).

4. Aneuploidy: This study does not directly address aneuploidy or trisomy pregnancies. However, the most significant predictors of PTB in this study were diabetes and PAPP-A. Diabetes and low PAPP-A are both associated with trisomy pregnancies, and trisomy pregnancies have increased risks of PTB. Consequently, this should be addressed/clarified in this manuscript.
We have added this to the limitations section of the discussion section in line with the reviewer’s suggestion.

5. Grammar: Please carefully review the manuscript at length for typos. Below are several that were identified on my review:
- A parenthesis is missing after "(Supplemental Table 2" on page 6.
- In the last paragraph on page 9, the first sentence should include an "s" after "other model."
- An extra parenthesis should be removed after "(logistic regression, Supplementary Figure 2))" and "(logistic regression, Supplementary Figure 3))" on page 10.
- "Table S8" should be renamed "Supplemental Table 8" to be consistent with the rest of the manuscript.

We thank the reviewer for drawing our attention to these errors. Our manuscript has now undergone additional editorial review, through which we have addressed these and other points.

6. PLOS authors have the option to publish the peer review history of their article ([what does this mean?](https://journals.plos.org/plosone/s/editorial-and-peer-review-process#loc-peer-review-history)). If published, this will include your full peer review and any attached files.


If you choose “no”, your identity will remain anonymous but your review may still be made public.


**Do you want your identity to be public for this peer review?** For information about this choice, including consent withdrawal, please see our [Privacy Policy](https://www.plos.org/privacy-policy).

Reviewer #1: **Yes:** Giuseppe Rizzo

Reviewer #2: **Yes:** Katelyn J Rittenhouse, MD

[NOTE: If reviewer comments were submitted as an attachment file, they will be attached to this email and accessible via the submission site. Please log into your account, locate the manuscript record, and check for the action link "View Attachments". If this link does not appear, there are no attachment files.]

While revising your submission, please upload your figure files to the Preflight Analysis and Conversion Engine (PACE) digital diagnostic tool, <https://pacev2.apexcovantage.com/>. PACE helps ensure that figures meet PLOS requirements. To use PACE, you must first register as a user. Registration is free. Then, login and navigate to the UPLOAD tab, where you will find detailed instructions on how to use the tool. If you encounter any issues or have any questions when using PACE, please email PLOS at [figures@plos.org](mailto:figures@plos.org). Please note that Supporting Information files do not need this step.
